# GLMYsymm: inferring symmetry categories of protein complexes from single sequences using persistent GLMY homology

**DOI:** 10.1093/bib/bbag449

**Published:** 2026-08-24

**Authors:** Jiaqi Zhai, Jingyan Li, Shing-Tung Yau, Xinqi Gong

**Affiliations:** School of Mathematics, Renmin University of China, No. 59 Zhongguancun Street, Haidian District, Beijing 100872, China; Beijing Institute of Mathematical Sciences and Applications, No. 544 Hefangkou Village, Huaibei Town, Huairou District, Beijing 101408, China; Yau Mathematical Sciences Center, Tsinghua University, No. 30 Shuangqing Road, Haidian District, Beijing 100084, China; School of Mathematics, Renmin University of China, No. 59 Zhongguancun Street, Haidian District, Beijing 100872, China

**Keywords:** homologous protein complexes, symmetry prediction, GLMY homology, single-sequence model, deep learning

## Abstract

Proteins often perform essential biological functions in the form of complexes. The assembly of protein complexes typically exhibits symmetry, which contributes to a more stable structural organization. Therefore, investigating structural symmetry is of great importance for protein complex structure prediction. However, existing methods for predicting protein structural symmetry are limited in number and generally show unsatisfactory accuracy. To address this issue, we propose GLMYsymm, a single-sequence–based model for symmetry prediction of homologous protein complexes. The key innovation of this work lies in the use of persistent Grigor’yan–Lin–Muranov–Yau (GLMY) homology to extract topological information from protein sequences, which is further integrated with the Evolutionary Scale Modeling 2 (ESM2) pretrained model to enable end-to-end symmetry prediction directly from sequence data. To the best of our knowledge, this is the first study to apply persistent GLMY homology to protein sequence feature extraction, without relying on structural information or multiple sequence alignment. Through extensive ablation and comparative experiments, we further demonstrate the effectiveness of GLMY homology–based topological sequence features for training deep learning models. Furthermore, the GLMYsymm framework outperforms existing sequence-based methods, achieving an improvement of approximately 0.32 in Macro area under the precision–recall curve (AUC-PR) over Seq2Symm and QUEEN models on the same test dataset. In the field of protein structure prediction, GLMYsymm can be used to assess the symmetry category of predicted protein structures, thereby assisting in protein structure quality evaluation, and can also serve as an important reference for stoichiometry prediction. The code and datasets for GLMYsymm are available at http://mialab.ruc.edu.cn/GLMYsymmServer/.

## Introduction

Symmetric quaternary structures of proteins are highly prevalent in nature [1]. Since protein structure determines function [2], structural symmetry enables the formation of more complex architectures through relatively simple assembly mechanisms, thereby facilitating more sophisticated biological functions [3]. In addition, protein symmetry contributes to enhanced structural stability [4], increased flexibility, conservation of genetic resources, and functional cooperativity [5]. Investigating protein symmetry is therefore essential for understanding the organizational principles and functional mechanisms of protein quaternary structures. Beyond its biological relevance, accurate symmetry characterization also provides important structural priors for computational tasks such as protein complex structure prediction and quality assessment.

Despite its importance, accurate characterization of protein symmetry remains challenging in practice. This is largely because experimentally determining protein quaternary structures remains costly and labor-intensive [6], and a large number of proteins still lack resolved structures. In contrast, protein sequence information is much easier to obtain and is far more abundant than experimentally determined structures [7, 8]. In recent years, deep learning–based computational methods for protein structure prediction have achieved remarkable progress. Methods such as AlphaFold2 [7] and AlphaFold3 [9] have reached accuracies exceeding 90% for monomeric protein structure prediction. However, there remains substantial room for improvement in the prediction of multimeric protein structures. Predicting protein structural symmetry using deep learning approaches has the potential to further enhance the accuracy of protein complex structure prediction.

To analyze protein symmetry, several computational methods have been developed. Existing approaches can generally be divided into structure-based and sequence-based methods. Structure-based methods, including SymD [10], CE-Symm [11], and DeepSymmetry [12], can detect symmetric residue pairs and symmetry axes, enabling the analysis of symmetry and pseudosymmetry in multimeric protein structures [13]. However, these methods require experimentally determined protein structures in order to perform geometric analysis, which limits their applicability.

Given the limited availability of experimentally resolved quaternary structures, sequence-based approaches have emerged as a promising alternative for symmetry prediction. Several deep learning–based sequence-driven methods have been proposed to predict the symmetry of homologous protein structures (e.g. Seq2Symm [14]) or to infer homologous protein assembly modes (e.g. QUEEN [15]). However, Seq2Symm relies on multiple sequence alignment (MSA) information, which increases computational cost, while QUEEN is unable to determine the intrinsic symmetry type of a protein complex.

**Figure 1:**
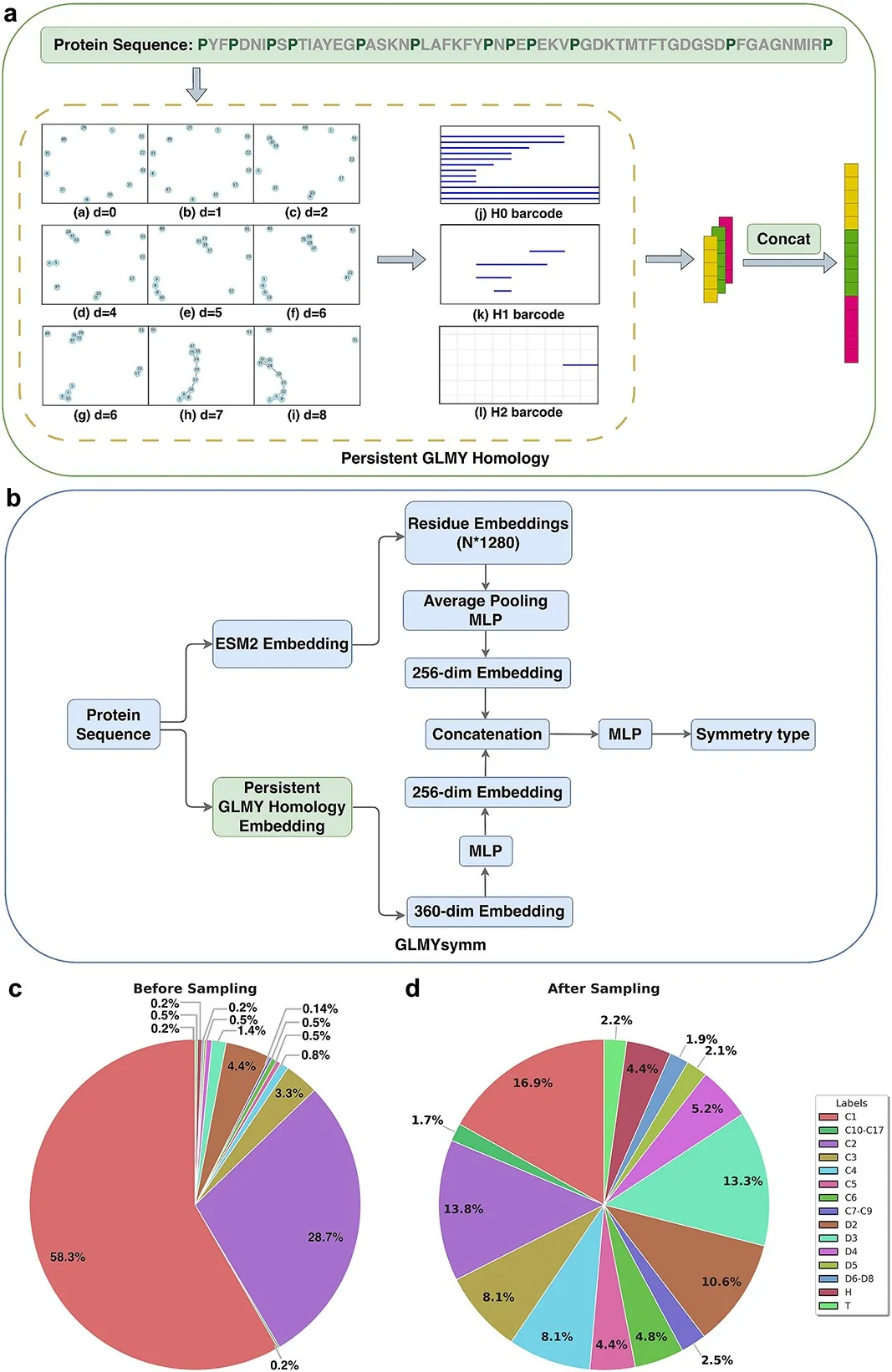
Overview of the GLMYsymm framework and data distribution. (a) Schematic illustration of persistent GLMY homology feature extraction. Within the dashed box, the left panels show the undirected graphs $\mathbf{G}(d_{i})$ constructed at relative distances *d=0,1, ⋯ ,8*, while the right panels present the barcodes corresponding to the birth and death of 0-dimensional, 1-dimensional, and 2-dimensional homology classes throughout the filtration process. Statistical features are extracted from the barcode diagram of each residue in the sequence, and these features are concatenated to form the persistent GLMY homology representation of the entire sequence. (b) Workflow of GLMYsymm. (c) Distribution of samples across different symmetry classes before sampling. (d) Distribution of samples across different symmetry classes after sampling. As shown in the figure, after oversampling and resampling, the data distribution becomes more balanced, which enables the model to better learn and attend to symmetry classes with limited sample sizes.

Therefore, there remains a need for a sequence-only framework that can directly infer intrinsic symmetry classes without relying on experimentally resolved structures or computationally expensive MSA information. To address this gap while further improving prediction accuracy for homologous protein complexes, we propose GLMYsymm, a method that predicts the symmetry of homologous protein complex structures by extracting sequence topological features using persistent GLMY homology. The overall workflow is illustrated in Fig. 1a and b. Given the strong correlation between protein homology and structural symmetry—namely, homologous proteins tend to exhibit symmetric structures, and most symmetric structures arise from homologous assemblies [16, 17]—this study focuses specifically on homologous protein complexes.

The main contributions of GLMYsymm are summarized as follows:

1. We use statistical features derived from persistent GLMY homology barcodes as topological features extracted from protein sequences to predict the symmetry category of protein complex structures. To the best of our knowledge, this is the first application of persistent GLMY homology to protein sequence feature extraction.
2. GLMYsymm relies solely on sequence information, without requiring structural data or MSA, enabling end-to-end prediction from protein sequence to symmetry.
3. By analyzing features extracted via persistent GLMY homology, we observe clear separability of sequence topological features across different symmetry classes, further demonstrating the relevance of sequence topology for protein symmetry prediction.
4. Through comparisons among different persistent GLMY homology feature extraction strategies, ablation studies, and benchmarking against existing methods, we demonstrate the effectiveness of persistent GLMY homology–derived sequence topological information for training deep learning models, providing guidance for future research in related problems.

In this study, we systematically evaluated multiple strategies for extracting sequence features using persistent GLMY homology and ultimately selected a configuration that computes only 1-mer features with a maximum filtration value of 8 and homology dimension 2. Under this setting, the model achieved a Macro AUC-PR of 0.5921. Statistical analysis of the extracted persistent GLMY homology features revealed that the one-dimensional Betti number features of 1-mers exhibit clear separability among different symmetry classes, confirming that sequence topological features derived from persistent GLMY homology enable the model to distinguish between different symmetry categories.

Furthermore, we compared GLMYsymm with two deep learning–based methods, Seq2Symm and QUEEN, for protein complex symmetry prediction. Using Macro AUC-PR as the evaluation metric, GLMYsymm outperformed both methods by 0.32. These results demonstrate that persistent GLMY homology features can more precisely capture sequence information, allowing GLMYsymm to achieve improved prediction performance for homologous protein symmetry without relying on structural data or MSA information.

In summary, GLMYsymm enables more accurate prediction of symmetry in homologous protein complexes. The sequence topological information extracted via persistent GLMY homology shows meaningful associations with protein symmetry, allowing the model to better learn symmetry-related features. GLMYsymm therefore provides a valuable methodological reference for future research in related areas.

## Datasets

In this study, the data were obtained from the Protein Data Bank (PDB) [18]. The detailed data processing procedure is described as follows.

Based on the symmetry annotations provided in the PDB database, protein structures were retrieved using the advanced search function [1]. Specifically, protein complexes exhibiting $C_{n}$ symmetry (*n =* 1–17), $D_{n}$ symmetry (*n =* 2–8, where $D_{1}$ is equivalent to $C_{2}$ symmetry), as well as H and T symmetries were collected. All selected structures were required to contain no more than 20 chains. This procedure yielded an initial set of 329 768 FASTA sequences.

Structures with $C_{n}$ (*n> 17*), $D_{n}$ (*n> 8*), I and O symmetry, or with more than 20 chains were excluded from this study. After applying the above filtering criteria, the number of structures belonging to these symmetry types was extremely limited (only a few or several dozen), and these structures are typically very large. Including them would introduce noise into the model and negatively affect learning efficiency.

To remove redundancy, MMseqs2 [19] was employed to cluster protein sequences. All sequences were clustered with a minimum sequence identity of 30% and an alignment coverage of at least 80% of the sequence length. Representative protein structures from each cluster were then selected to construct the dataset. This procedure effectively prevents data leakage during model training. After redundancy removal, the dataset was split into training, validation, and test sets with a ratio of 8:1:1.

The number of samples varies substantially across different symmetry classes, resulting in severe class imbalance that significantly degrades model performance. To address this issue, sampling strategies were applied to the training dataset. Specifically, samples labeled as $C_{1}$ were undersampled to 60% of their original size; $C_{2}$ samples were left unchanged; $C_{3}$ and $D_{2}$ samples were oversampled by a factor of 5; and all remaining symmetry classes, which had very limited sample sizes, were oversampled by a factor of 20. This strategy was designed to increase the number of samples in extremely small classes to approximately 1000, bringing them to the same order of magnitude as the larger classes. The data distributions before and after sampling are shown in Fig. 1c and d.

Some protein monomer sequences were associated with multiple symmetry labels, meaning that the same single-chain protein could assemble into complexes with different symmetry types. For example, protein 3MSR can form assemblies with $C_{1}$, $C_{2}$, $C_{3}$, and $D_{3}$ symmetries. Since such multi-label samples may adversely affect model performance and account for only a small fraction of the dataset, all multi-label data were removed.

Because high-order symmetric protein complexes are substantially underrepresented in current public databases, several symmetry classes with extremely limited sample sizes were merged to alleviate severe class imbalance. The label merging strategy was primarily motivated by the following three considerations.

(1) To mitigate the severe class imbalance caused by the extreme scarcity of samples in several high-order symmetry classes.
(2) To ensure that each merged category contains a sufficient number of training samples while preserving the continuity of symmetry orders and structural similarity, thereby improving the stability and generalization ability of the model. Following this principle, $C_{7}$–$C_{9}$, $C_{10}$–$C_{17}$, and $D_{6}$–$D_{8}$ were merged into three independent categories.
(3) The adopted merging strategy was also guided by the category definition used in Seq2Symm. Specifically, Seq2Symm similarly merges $C_{7}$–$C_{9}$, $C_{10}$–$C_{17}$, and high-order dihedral symmetry classes into broader categories. Therefore, the category mapping adopted in this study is highly consistent with that of Seq2Symm. Furthermore, all comparisons with Seq2Symm and QUEEN were conducted using the same dataset partition and unified category mapping framework, ensuring the fairness and consistency of the reported results.

Consequently, $C_{7}$–$C_{9}$, $C_{10}$–$C_{17}$, and $D_{6}$–$D_{8}$ were treated as three composite categories in the final classification task. The sample counts of the original symmetry classes before merging, together with the corresponding label merging strategy, are summarized in Supplementary Table S3 of the Appendix.

## Results

### GLMYsymm overview

GLMYsymm is a deep learning model that predicts the symmetry of homologous protein complexes by extracting sequence topological features using persistent GLMY homology and integrating them with representations derived from the ESM2 large-scale pretrained language model. The input to the model is the single-chain amino acid sequence of a protein whose symmetry is to be predicted, and the output is the predicted symmetry class.

The overall workflow of GLMYsymm is as follows. First, for each input single-chain sequence, persistent GLMY homology is used to extract 360-dimensional sequence topological features, which are subsequently reduced to 256 dimensions using a multilayer perceptron (MLP). In parallel, the ESM2 pretrained language model is applied to compute sequence embeddings for the protein, and the resulting residue-level features are averaged across the residue dimension to obtain a global sequence representation for the single chain. This representation is also projected to 256 dimensions using an MLP. The two feature representations are then concatenated and fused through an MLP-based architecture. Finally, a prediction head is applied to output the symmetry class of the protein complex.

Additional methodological details are included in the Appendix (Methods section), and a schematic illustration of the model architecture is shown in Fig. 1b.

### Correlation analysis between 1-mer homology features and protein symmetry

**Figure 2:**
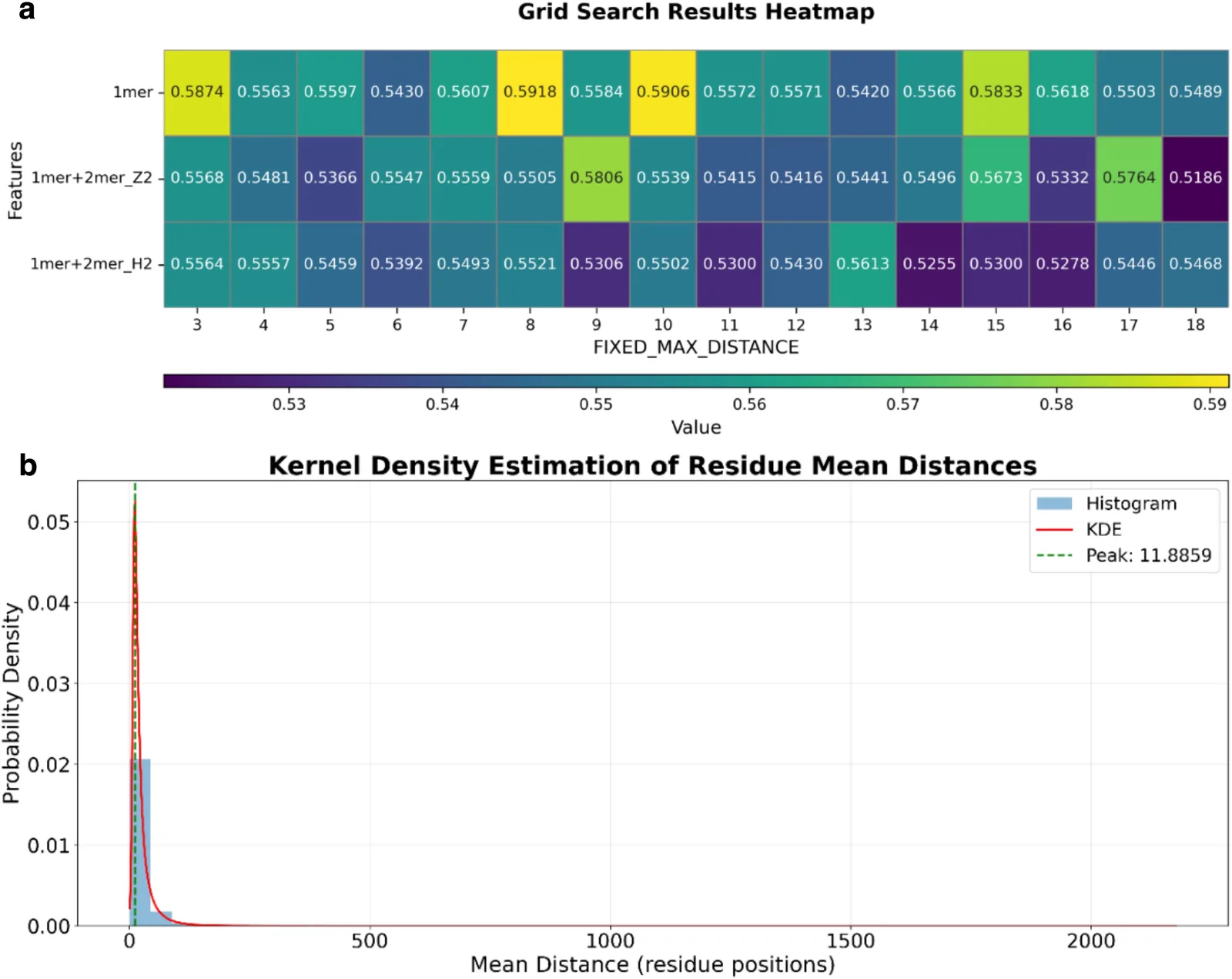
Feature and hyperparameter selection. (a) Each row corresponds to a different feature extraction strategy, and each column corresponds to a specific value of the maximum filtration distance. The numbers shown indicate the Macro AUC-PR of the model on the training and Validation sets for the corresponding parameter combination. Higher values indicate better predictive performance. (b) Distribution of the average distance from each residue to its two nearest identical residues. The blue bars represent the normalized distance distribution histogram, the red curve shows the Gaussian KDE, and the green dashed line indicates the peak of the probability density function at 11.89 residues.

We construct persistent GLMY homology features from protein sequences using both individual residues (1-mer) and residue pairs (2-mer) to capture topological information. The maximum filtration distance is a key parameter affecting feature extraction. We compared models using only 1-mer features with those using both 1-mer and 2-mer features, and performed a grid search over the “FIXED_MAX_DISTANCE” parameter (Fig. 2). Based on these results, we adopt 1-mer features as the final topological representation. Further details are provided in the Supplementary Sections “Selection of 1-mer and 2-mer Features” and “Selection of Maximum Filtration Distance Parameter.”

We performed a systematic statistical analysis of the 1-mer persistent GLMY features to investigate whether there exist stable and interpretable topological differences among different protein symmetry categories. A differential scoring function, *score*, was introduced for this purpose (for details, see the “Differential Scoring” subsection of the Appendix).

**Figure 3:**
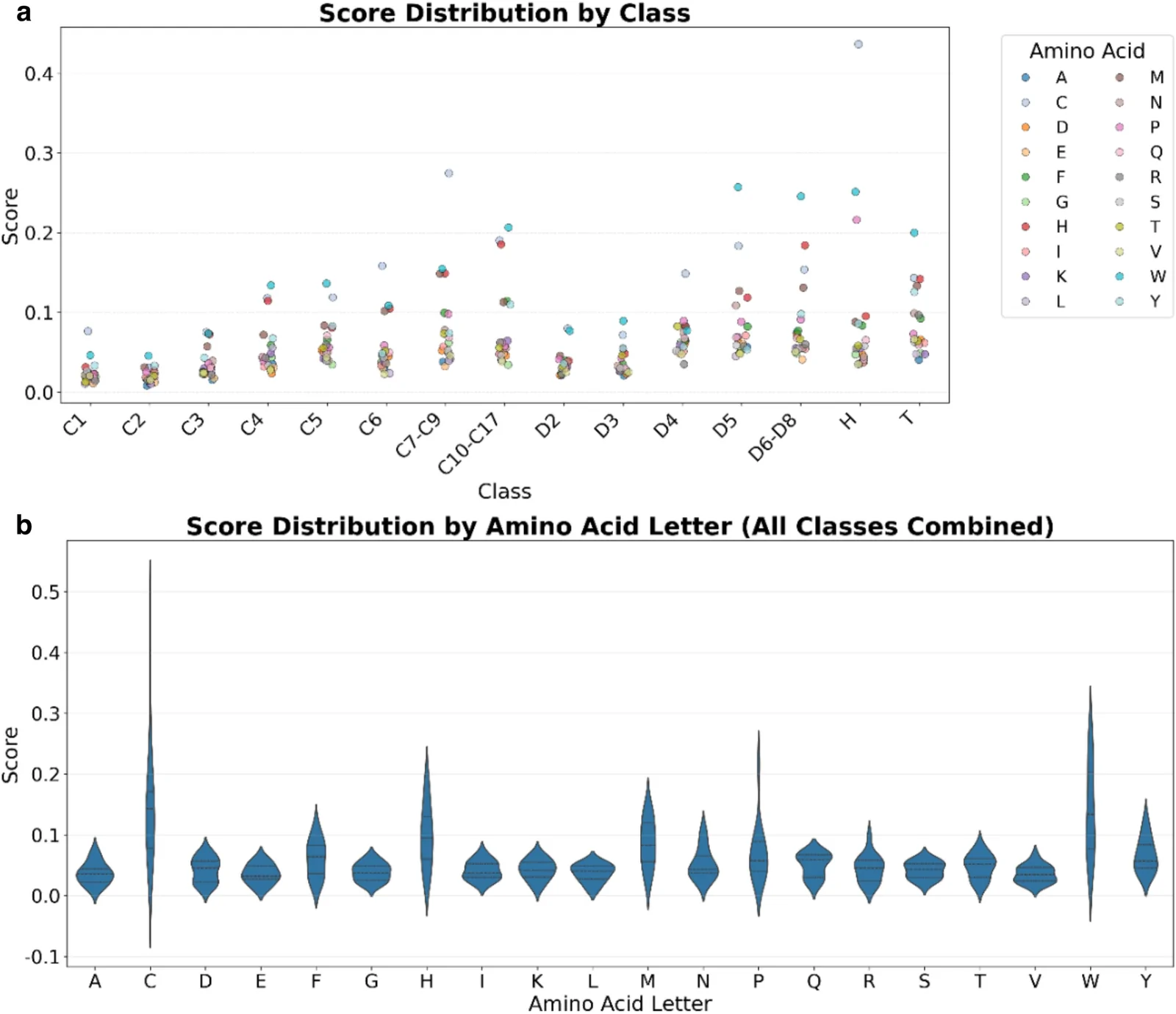
Distributions of *score* values. (a) Strip plot of *score* values for 20 standard amino acids across different symmetry categories. (b) Violin plot of *score* distributions for each amino acid across all symmetry categories.

For each residue type, *score* values were calculated across all symmetry categories. The results are visualized using strip plots and violin plots in Fig. 3a and b, with detailed numerical values provided in Supplementary Table S2 in the Appendix.

From the strip plot, the following observations can be made:

1. Low or non-symmetric categories (e.g. $C_{1}, C_{2}$) show almost no significant residue-specific $\beta _{1}$ signal, with topological structures being dispersed and lacking stable repetitive patterns.
2. Mid-to-low order rotational symmetries ($C_{3}$–$C_{6}$) already exhibit certain heterogeneity at the sequence level.
3. High-order rotational symmetries ($C_{7}$–$C_{9}$, $C_{10}$–$C_{17}$) are strongly dominated by a few residues (e.g. C, H, M, W), presenting highly concentrated and long-lived loops in 1-mer $\beta _{1}$ homology.
4. Compared with the same-order $C_{n}$ symmetries, the corresponding $D_{n}$ categories still retain strong $\beta _{1}$ signals. However, due to the introduction of mirror symmetry, the residue combinations become more complex, and the topological features are more dispersed across scales. This phenomenon constitutes a key topological evidence distinguishing *C* and *D* symmetries.
5. Extremely high-symmetry categories such as H and T exhibit nearly ”lattice-like” residue distance patterns, corresponding to extremely concentrated $\beta _{1}$ topological signals.

From the violin plot, it is evident that residues C, H, M, P, and W show large differences in their score values across different symmetry types, serving as sequence topological fingerprints that distinguish protein complexes with different symmetries. These highly discriminative residues possess distinctive biochemical properties. For example, C (Cysteine) can form disulfide bonds, which play critical roles in protein folding and oligomer stability. H (Histidine), whose acid–base properties are close to physiological pH, is frequently enriched in functionally important regions. P (Proline), owing to its unique cyclic side chain, imposes strong conformational constraints on the local backbone and secondary structure formation. W (Tryptophan) and M (Methionine) are highly hydrophobic residues that often participate in the formation of protein cores or protein–protein interaction interfaces. These biochemical constraints may cause the relative positional distributions of such residues along protein sequences to deviate from randomness and instead be jointly shaped by protein folding, oligomeric assembly, and functional requirements. Since different symmetry classes are associated with distinct assembly architectures and interface organizations, the distribution patterns of these residues within sequences may also vary accordingly, thereby giving rise to discriminative GLMY topological features. This indicates that the persistent GLMY homology features extracted from sequences are associated with protein structural symmetries; specifically, the 1-mer persistent GLMY homology $\beta _{1}$ features are significantly separable across different symmetry classes.

The persistent GLMY homology features enable the model architecture to better learn the topological differences induced by the relative positional distributions of identical residues in sequences across different symmetry types. These results demonstrate the meaningfulness and interpretability of extracting sequence topological features.

### Ablation study

**Figure 4:**
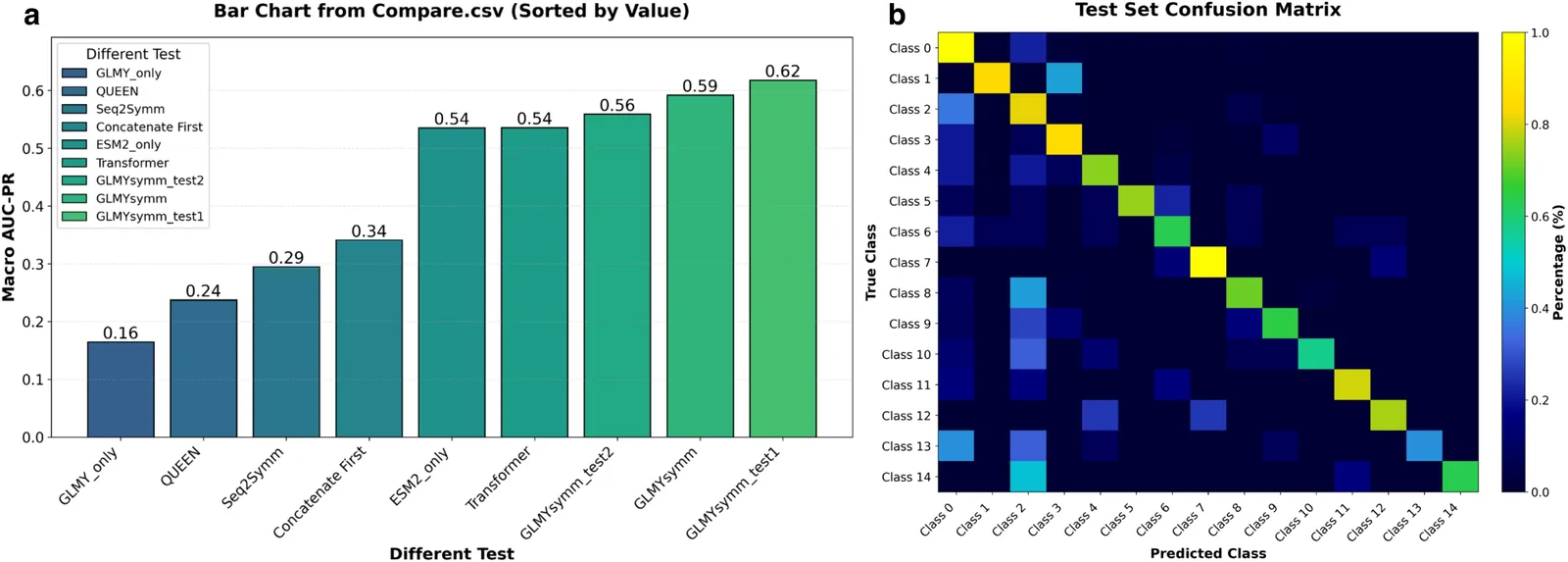
GLMYsymm test set results for different model variants and the corresponding confusion matrix. (a) In the bar chart, the x-axis represents different tested models: “GLMY_only” indicates the model using only GLMY features; “QUEEN” and “Seq2Symm” denote the baseline methods compared with GLMYsymm in this study; “Concatenate First” represents the model where persistent GLMY features and ESM2 features are first concatenated before prediction; “ESM2_only” indicates the model using only ESM2 features; “Transformer” denotes the variant where the MLP architecture in GLMYsymm is replaced with a Transformer; “GLMYsymm_test2” shows GLMYsymm performance on the test set after removing samples appearing in the QUEEN training set; “GLMYsymm” is the proposed model; “GLMYsymm_test1” shows GLMYsymm performance after removing samples appearing in the Seq2Symm training set. The models are ordered by Macro AUC-PR scores from lowest to highest. (b) The confusion matrix illustrates the classification performance of GLMYsymm on the test set. The color intensity indicates the percentage of samples of each true class predicted as each target class, ranging from dark blue (low percentage) to yellow (high percentage). Diagonal elements correspond to correctly classified samples, while off-diagonal elements show the distribution of misclassifications.

To assess the importance of different features and module designs in the GLMYsymm model, we conducted ablation experiments by removing certain features, replacing the feature fusion module, and adjusting the feature concatenation strategies. These experiments systematically evaluated the impact of each modification on the model’s performance on the test set, thereby validating the rationality and effectiveness of the overall model architecture. The results of these experiments are shown in Fig. 4. Furthermore, an additional ablation study was performed to evaluate the effect of the training-set oversampling strategy on class-imbalanced classification, and the detailed results are presented in Appendix D in the Supplementary Material.

#### Evaluation of individual feature types: ESM2 and persistent GLMY homology

To assess the contribution of GLMY homology-based sequence topology features to model performance, we first evaluated the model using single-feature inputs. Specifically, we constructed a model that only utilizes ESM2-derived sequence embeddings as input. Under this setting, with only a single feature type, the model employing an MLP classifier achieved a Macro AUC-PR of 0.5354 on the test set.

Subsequently, we constructed a model that uses only persistent GLMY features, also employing an MLP as the prediction network. This model achieved a Macro AUC-PR of 0.1647 on the test set.

Building upon these results, we further combined ESM2 features and persistent GLMY homology features as input, while keeping the prediction head architecture unchanged (still using an MLP). The feature-fusion model achieved a Macro AUC-PR of 0.5921 on the test set, demonstrating a clear improvement over models using either single feature alone.

These results indicate that persistent GLMY homology features alone are insufficient to fully capture the semantic information of protein sequences. However, when used as a structured topological complement to ESM2 sequence embeddings, they effectively enhance the model’s discriminative capability and further improve overall prediction performance. This finding validates the necessity and effectiveness of incorporating GLMY homology features as auxiliary information in the GLMYsymm model.

#### Comparison between MLP and transformer architectures

In the previous experiments, we demonstrated that the combination of ESM2 features and persistent GLMY homology features yields the best predictive performance. Building upon this, we further compared two different feature fusion architectures, namely MLP and Transformer, to evaluate the impact of the fusion strategy on model performance.

In both comparative experiments, the input features consisted of the concatenation of GLMY features and ESM2 features, with a shared linear layer used as the prediction head. Under the MLP-based fusion architecture, the model achieved a Macro AUC-PR of 0.5921 on the test set.

Subsequently, we replaced the feature fusion module with a Transformer architecture to model potential interactions between the two feature modalities. Specifically, the concatenated fixed-length feature vector (512 dimensions) was reshaped into a sequence of 8 tokens, where 4 tokens originated from GLMY features and 4 tokens from ESM2 features. This tokenization was chosen to balance model expressivity and computational complexity: using too many tokens would significantly increase computational cost, while the main goal of introducing the Transformer in this study was to model potential interactions and limited relative positional information between the two feature types. In this setting, the Transformer module contained 8 attention heads and 2 encoder layers. The resulting model achieved a Macro AUC-PR of 0.5359 on the test set.

These results indicate that, for the current task, there are no significant higher-order interactions between ESM2 and GLMY features. In contrast, the MLP architecture is more effective in preserving and integrating the discriminative information from both feature types, thereby achieving superior predictive performance. This finding suggests that, given the current feature representations and task scale, a simple and stable MLP is more suitable as the feature fusion module.

#### Comparison of feature concatenation strategies

The previous experiments demonstrated that using both persistent GLMY features and ESM2 features can significantly improve model performance. However, the order of feature concatenation and dimensionality reduction during multi-source feature fusion remains an important consideration. To investigate this, we conducted comparative experiments evaluating different feature concatenation strategies.

We mainly considered two fusion strategies. The first strategy, “reduce-then-concatenate,” involves independently reducing the dimensionality of GLMY features and ESM2 features to 256 dimensions via separate linear layers or MLPs, followed by concatenation into a 512-dimensional representation. This representation is then fed into the MLP network and prediction head to perform symmetry classification. Under this setting, the model achieved a Macro AUC-PR of 0.5921 on the test set.

The second strategy, “concatenate-then-reduce,” involves first concatenating GLMY and ESM2 features directly and then compressing the high-dimensional combined features to 512 dimensions via an MLP before passing them through the prediction head. To ensure comparable network depth with the first strategy, the number of MLP layers in this scheme was doubled. However, the resulting model only achieved a Macro AUC-PR of 0.3410 on the test set, indicating a substantial performance drop.

These results indicate that for features originating from different sources with distinct statistical properties, the “reduce-then-concatenate” strategy more effectively preserves the discriminative information of each feature type, leading to superior predictive performance. Therefore, this feature concatenation approach was adopted as the final feature fusion scheme in the GLMYsymm model.

Overall, the ablation experiments demonstrate that GLMY features provide complementary information for sequence representation, that MLP-based fusion is more effective than Transformers for the current task in preserving discriminative information, and that reducing feature dimensions individually before concatenation yields better performance. The GLMYsymm architecture integrates the most effective and reasonable combination of feature types and model components.

### Effectiveness of persistent GLMY homology features with traditional machine learning classifiers

To further evaluate whether the performance improvement originates from the proposed feature representation itself rather than solely from the downstream neural network architecture, we additionally introduced four traditional machine learning classifiers, including logistic regression (LR), random forest (RF), support vector machine (SVM), and Extreme Gradient Boosting (XGBoost). Each classifier was trained and evaluated using ESM2 features, persistent GLMY Homology features, and their fused representations. The results are summarized in Table 1.

**Table 1:** Performance comparison of traditional machine learning classifiers using ESM2 features, persistent GLMY homology features, and their fusion on the test set.

Classifier	Feature representation	Macro-F1	Macro-AUC-PR
LR	ESM2	**0.4620**	0.4775
	Persistent GLMY homology	0.0896	0.1117
	ESM2 + persistent GLMY homology	0.4447	**0.4844**
RF	ESM2	0.1920	0.4952
	Persistent GLMY homology	0.1424	0.2141
	ESM2 + persistent GLMY homology	**0.2397**	**0.4996**
SVM	ESM2	0.5517	0.5466
	Persistent GLMY homology	0.1822	0.1989
	ESM2 + persistent GLMY homology	**0.5740**	**0.5500**
XGBoost	ESM2	0.5090	0.5257
	Persistent GLMY homology	0.1891	0.1930
	ESM2 + persistent GLMY homology	**0.5186**	**0.5290**

Bold values indicate the best performance among the compared models for each metric0

As shown in Table 1, when only persistent GLMY homology features were used, all four classifiers achieved non-zero classification performance, with Macro-F1 ranging from 0.0896 to 0.1891 and Macro AUC-PR ranging from 0.1117 to 0.2141. Although these results were consistently inferior to those obtained using ESM2 features, this observation is expected. ESM2 is a protein language model pretrained on massive protein sequence databases and is therefore capable of capturing rich evolutionary, structural, and functional information from protein sequences. In contrast, persistent GLMY homology represents proteins solely through topological patterns induced by amino acid distributions within a single sequence. Consequently, persistent GLMY homology alone is not expected to match the overall predictive performance of ESM2.

More importantly, the fused representation consistently achieved higher Macro AUC-PR than ESM2 alone across all four traditional machine learning classifiers. Specifically, the Macro AUC-PR increased from 0.4775, 0.4952, 0.5466, and 0.5257 to 0.4844, 0.4996, 0.5500, and 0.5290 for LR, RF, SVM, and XGBoost, respectively. Since Macro AUC-PR is the primary evaluation metric adopted in this study, these results indicate that persistent GLMY homology provides complementary information beyond that captured by ESM2 embeddings.

In terms of Macro-F1, the fused representation outperformed ESM2 alone in RF, SVM, and XGBoost. Specifically, the Macro-F1 score improved from 0.1920 to 0.2397 for RF, from 0.5517 to 0.5740 for SVM, and from 0.5090 to 0.5186 for XGBoost. By contrast, the Macro-F1 score of the fused representation in LR was slightly lower than that of ESM2 alone (0.4447 versus 0.4620), although its Macro AUC-PR still improved. This observation suggests that the complementary relationship between ESM2 and persistent GLMY homology is not merely a linear combination, but is more likely associated with non-linear feature interactions. Consequently, classifiers with stronger non-linear modeling capabilities are able to exploit the additional information provided by persistent GLMY homology more effectively.

Notably, although persistent GLMY homology alone achieved substantially lower performance than ESM2, incorporating it into the fused representation consistently improved Macro AUC-PR across all four classifiers. This finding indicates that the topological information captured by persistent GLMY homology is not fully contained within ESM2 embeddings and can therefore serve as an independent and complementary source of information for protein symmetry category prediction.

Overall, the results obtained using traditional machine learning classifiers demonstrate that persistent GLMY homology provides additional and complementary information beyond protein language model embeddings. Furthermore, this benefit is not restricted to a specific deep learning architecture. Therefore, the performance gain achieved by GLMYsymm can be attributed not only to the modeling capability of the downstream architecture but also to the informative topological representations captured by the proposed persistent GLMY homology features.

### Comparison with other methods

#### Comparison with Seq2Symm

Seq2Symm, proposed by the David Baker group [14], is a deep learning method for predicting the symmetry of homomeric protein complexes based on protein sequence information. This method mainly leverages sequence embeddings extracted by ESM2 and MSA features, followed by a classifier head to predict the symmetry type of protein complexes. In the original study, Seq2Symm achieved a Macro AUC-PR of 0.47 on its self-constructed test set.

To ensure a fair comparison, we evaluated Seq2Symm and GLMYsymm on a unified test set. Specifically, to avoid potential data leakage, we first removed any samples from the test set that appeared in the original Seq2Symm training set. Subsequently, predictions were performed on the reconstructed test set, and the Macro AUC-PR and Macro Precision metrics were calculated for both methods.

The results show that Seq2Symm achieved a Macro AUC-PR of 0.2950 and a Macro Precision of 0.4337 on this test set. In contrast, GLMYsymm achieved a Macro AUC-PR of 0.6179 and a Macro Precision of 0.6144, demonstrating a substantial improvement over Seq2Symm.

#### Comparison with QUEEN

QUEEN [15] is a method designed to predict the quaternary assembly of homomeric protein complexes from single-chain sequences. Considering that homomeric complexes in practice mostly exhibit symmetry, the prediction task of QUEEN aligns closely with the symmetry prediction task addressed by GLMYsymm. Therefore, we conducted a comparative evaluation of QUEEN and GLMYsymm on the same unified test set.

Similarly, to prevent data leakage, we first removed any samples in the test set that appeared in the original QUEEN training set. As QUEEN predicts the copy number of chains in the complex rather than directly predicting symmetry types, we performed label mapping for the comparison. Specifically, we retained all $C_{n}$ (*n = 1,2,3,4,5,6,7,8,10,12,14,24*) and $D_{n}$ (n = 1,2,3,4,5,6,7) samples, mapping $C_{n}$ to a copy number of *n* and $D_{n}$ to *2n*. Symmetry types that could not be uniquely mapped (e.g. helical H symmetry corresponding to multiple copy numbers, and tetrahedral T symmetry corresponding to 12 or 24 copies) were excluded from the test set.

After this preprocessing, predictions were made using both methods on the unified test set, and Macro AUC-PR and Macro Precision were computed. QUEEN achieved a Macro AUC-PR of 0.2372 and a Macro Precision of 0.6161, while GLMYsymm achieved 0.5591 and 0.6355, respectively.

Overall, these comparative experiments demonstrate that GLMYsymm outperforms both Seq2Symm and QUEEN on the unified test set, particularly in terms of the Macro AUC-PR metric, highlighting the effectiveness and robustness of the proposed method for homomeric complex symmetry prediction.

#### Comparison with handcrafted sequence features

To further evaluate the effectiveness of the features extracted by persistent GLMY homology and investigate whether they provide information beyond simple sequence-based statistics, we constructed three representative handcrafted sequence feature baselines: (i) k-mer frequency features (8420 dimensions), which describe local sequence composition patterns; (ii) autocorrelation features (720 dimensions), which characterize residue correlations based on physicochemical property indices from the AAindex database; and (iii) residue spacing histogram features (400 dimensions), which capture the distribution of distances between residues of the same amino acid type. For comparison, the proposed persistent GLMY homology features were derived from path homology persistence information and represented by a 360-dimensional feature vector.

To ensure a fair comparison of the intrinsic representational capabilities of different feature types, all experiments were conducted using the same dataset split and the same RF classifier, with only the input features being replaced during training and testing.

Table 2 presents the classification results of different feature representations on the test set. The results show that the GLMY homology features achieved the best performance across all evaluation metrics. Although the k-mer frequency, autocorrelation, and residue spacing histogram features capture statistical information related to amino acid composition, physicochemical property correlations, and residue spacing distributions, respectively, the topological features extracted by persistent GLMY homology exhibited stronger discriminative power. Notably, despite the k-mer frequency features having a dimensionality of 8420, substantially higher than the 360 dimensions of the GLMY features, their performance remained inferior to that of GLMY homology. This suggests that the information captured by GLMY features is not merely high-dimensional statistical information, but rather reflects more informative topological organizational patterns within protein sequences. These results indicate that persistent GLMY homology provides complementary information beyond simple sequence statistical descriptors, thereby improving the prediction of protein symmetry classes.

**Table 2:** Comparison of GLMY homology and handcrafted sequence features using the same RF classifier.

Feature	Bal.Acc	Macro-F1	Macro-Recall	Macro AUC-PR
k-mer frequency	0.1154	0.1347	0.1154	0.1929
Autocorrelation	0.0894	0.0964	0.0894	0.1368
Residue spacing histograms	0.1073	0.1259	0.1073	0.1920
Persistent GLMY homology	**0.1200**	**0.1424**	**0.1200**	**0.2141**

*Note:* Bal.Acc, balanced accuracy; Macro-F1, macro-averaged F1-score; Macro-Recall, macro-averaged recall; Macro AUC-PR, macro-averaged area under the precision–recall curve. Bold values indicate the best performance among the compared models for each metric.

### Model generalization

**Figure 5:**
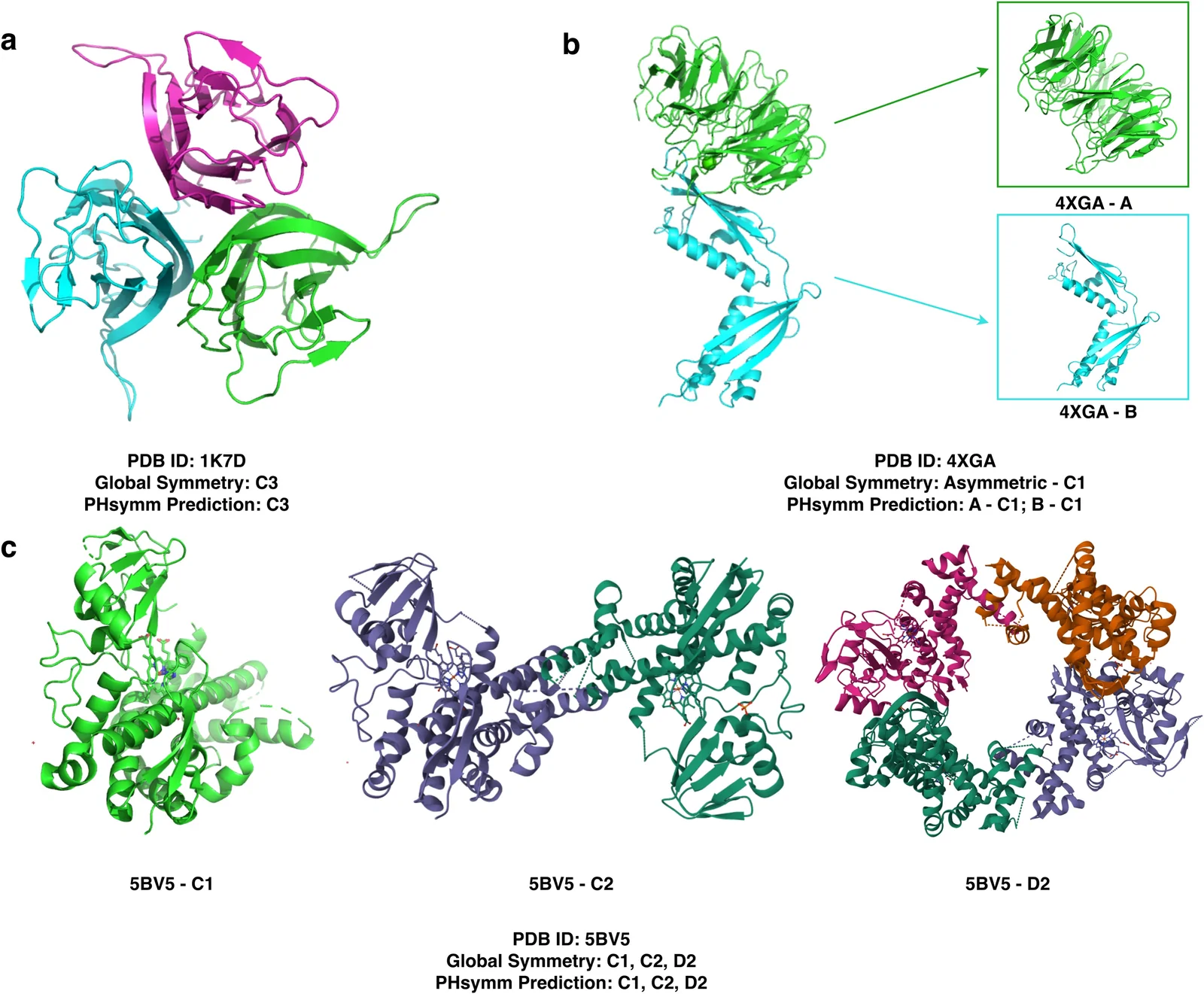
Generalization performance of GLMYsymm. (a) Structural representation of the homologous soluble protein 1K7D. The symmetry type predicted by GLMYsymm is consistent with the experimentally determined symmetry, both being C3. (b) Structural representation of the heterologous insoluble protein 4XGA. The overall structure is asymmetric (C1). When each chain is predicted separately using GLMYsymm, the predicted symmetry type for both chains is C1, which is consistent with the actual structure. (c) The protein 5BV5 can form three symmetry states: C1, C2, and D2. The top three predicted symmetry types by GLMYsymm are C1, C2, and D2, which are consistent with the experimentally observed possibilities.

To validate the generalization capability of GLMYsymm, we conducted predictions on several proteins of different types that were not included in the dataset and obtained favorable results. The results are illustrated in Fig. 5, and a detailed analysis can be found in the “Model Generalization” section of the Supplementary Materials.

## Discussion

In this study, we propose GLMYsymm, which, to the best of our knowledge, is the first model to apply persistent GLMY homology—a mathematical framework from topological data analysis—to the extraction of topological features from protein sequences. Through ablation studies and comparisons with Seq2Symm and QUEEN, we demonstrate that persistent GLMY homology–based sequence features capture richer information, enabling the model to more effectively learn sequence characteristics and predict protein symmetry. Nevertheless, several challenges remain to be addressed.

At present, homology-based approaches for sequence analysis have been successfully applied to small molecules such as DNA and RNA [20]. Because these sequences are relatively simple and consist of only four nucleotide types (A, T, C, and G), the number of possible symbol combinations is limited, making it feasible to compute higher-order k-mer features (i.e. combinations of multiple symbols). In contrast, protein sequences contain 20 different residue types, and the number of possible combinations grows exponentially, leading to a substantial increase in computational cost. How to efficiently compute higher-order mer features and systematically evaluate their impact on protein sequence representation remains an important direction for future research.

Severe class imbalance in the dataset negatively affects model performance. To mitigate this issue, undersampling, oversampling, and label merging were employed during data preprocessing, and Macro AUC-PR was adopted as an appropriate evaluation metric. In particular, several high-order symmetry groups (e.g. C7–C9, C10–C17, and D6–D8) were merged into broader categorical labels to improve data balance and statistical robustness. Consequently, GLMYsymm should be interpreted as predicting symmetry order categories rather than resolving exact symmetry orders within these merged intervals. Nevertheless, the scarcity of experimentally resolved structures for many symmetry classes remains an objective limitation. Additional data will be required in future studies to enable finer-grained symmetry discrimination and further improve model robustness and predictive performance.

It should be noted that although C1 is included among the predicted categories in this study, it is treated only as a category label within the unified symmetry classification framework and should not be interpreted as an explicit indication that a protein cannot form homologous complexes or that it exists solely as a monomer. For example, the protein with PDB ID 3MSR can adopt multiple symmetry classes, including C1, C2, C3, and D3, indicating that C1 primarily reflects a specific symmetry configuration under a particular assembly state rather than serving as an absolute marker of whether a protein is capable of forming homologous oligomeric complexes. The specific manner in which protein monomers assemble belongs to the problem of stoichiometry prediction, which will be an important direction of our future work and will be further extended to the study of heteromeric complexes.

Despite these challenges, which are difficult to resolve in the short term, the feature extraction strategy based on persistent GLMY homology used in GLMYsymm provides valuable methodological insights for protein symmetry prediction and related research areas. The proposed approach demonstrates the potential of topological sequence representations and offers meaningful guidance for future studies in this domain.

## Conclusion

Accurate prediction of protein complex symmetry is of great importance for understanding the assembly mechanisms of multimeric protein structures, functional analysis, and structure quality assessment. Given the strong correlation between protein homology and structural symmetry, this study focuses on homologous protein complexes and systematically investigates the problem of symmetry prediction for homologous protein assemblies. We propose GLMYsymm, a single-sequence model that predicts the symmetry categories of homologous protein complexes by extracting sequence topological features using persistent GLMY homology and integrating them with ESM2-derived sequence features.

In terms of feature construction, we systematically evaluated multiple parameter configurations and feature extraction strategies for persistent GLMY homology. The best performance was achieved using 1-mer–based sequence topological features with a maximum filtration distance of 8, incorporating homological information in dimensions 0 and 1 together with the dimension of the 2-cycle group $c_{2}=\dim \ker (\partial _{2})$. Furthermore, we analyzed the distribution of 1-mer persistent GLMY homology $\beta _{1}$ features corresponding to different residue types across symmetry classes. The statistical results demonstrate clear separability of these features among different symmetry categories, revealing an intrinsic relationship between sequence topology and complex symmetry from a topological perspective. This observation provides further insight into the effectiveness of persistent GLMY homology features in protein sequence analysis.

Through a series of ablation studies, we determined the final architecture of GLMYsymm. Specifically, persistent GLMY homology features and ESM2 features are first reduced in dimensionality independently, then concatenated and fused through an MLP-based architecture, followed by a prediction head to determine the symmetry category. Experimental results indicate that this design effectively integrates semantic sequence information with topological structural information while maintaining model stability.

Under a unified test set and strict prevention of data leakage, we compared GLMYsymm with two representative methods, Seq2Symm and QUEEN. The results show that GLMYsymm achieves an improvement of approximately 0.32 in Macro AUC-PR, demonstrating its superior performance and strong generalization ability in predicting the symmetry of homologous protein complexes.

The main innovations of GLMYsymm lie in two aspects. First, this work is the first to introduce persistent GLMY homology from mathematical topology into protein sequence analysis, enabling the extraction of sequence information from a topological perspective and its successful application to protein symmetry category prediction. Second, GLMYsymm operates entirely on single-sequence input, without relying on protein three-dimensional structural information or MSA, thereby substantially reducing data requirements and computational cost while maintaining strong predictive performance. These characteristics endow GLMYsymm with significant extensibility and practical applicability, and provide new perspectives and methodologies for future research on topological sequence modeling and structure–function prediction in bioinformatics.

**Key Points**

- In this work, we propose GLMYsymm, a novel computational framework for predicting the symmetry categories of protein complexes directly from single sequences.
- We introduce persistent GLMY homology into protein sequence analysis and systematically demonstrate its effectiveness in capturing topological features relevant to symmetry prediction. To the best of our knowledge, this is the first study to employ topological methods based on directed graphs for protein sequence analysis.
- We further perform a correlation analysis between 1-mer homology features and protein symmetry, revealing interpretable topological differences among distinct symmetry classes.
- GLMYsymm operates entirely on single-sequence input, without requiring structural information or MSA, significantly reducing data dependency and computational cost while maintaining strong predictive performance.

## Supplementary Material

Web_Material_bbag449

## Data Availability

All data used in this study, including symmetry labels and dataset splits, are available for download from the “Source Code” section of the website at http://mialab.ruc.edu.cn/GLMYsymmServer/. The source code for the model architecture and the corresponding configuration files are available in the ”Source Code” section of the website at http://mialab.ruc.edu.cn/GLMYsymmServer/. Detailed instructions for model usage are provided in the README file.
