# Supplementary material for: GLMYsymm: inferring symmetry categories of protein complexes from single sequences using persistent GLMY homology
Source: Brief Bioinform. 2026 Aug 24;27(4):bbag449. doi: 10.1093/bib/bbag449 (PMC13502095; doi:10.1093/bib/bbag449)
Supplement: Supplementary file 1 [file web_material_bbag449.zip › Appendix_GLMYsymm_bbag449.pdf]

Appendix

Appendix A Methods

In this work, we propose GLMYsymm, a method that leverages GLMY homology to extract topological features from protein sequences and integrates these with ESM2-derived sequence features to predict the symmetry type of homologous protein complexes. The overall model workflow is illustrated in Figure 1**B** in the main text. Below, we provide a detailed description of the feature extraction methods and the components of each module in the model.

A.1 Exploration of GLMY homology–Based Feature Extraction Methods

GLMY homology is an algebraic topology–based framework specifically designed to characterize the topological structure of directed graphs. By defining boundary operators on directed paths, GLMY homology constructs chain complexes that capture higher-order connectivity patterns induced by directionality in directed networks. In GLMYsymm, GLMY homology is employed to extract topological features from protein sequences. The results of all feature extraction strategies explored in this study are summarized in Figure 1**A** in the main text, and the detailed methodologies and corresponding analyses are described as follows.

A.1.1 Construction and Selection of Relative-Position–Based Persistent Homology Features

Modeling Method

For each type of residue or residue pair in a protein sequence, we denote it by the symbol π. All occurrences of π in the sequence are identified and indexed as $\pi_{k_1}, \pi_{k_2}, \ldots, \pi_{k_n}$, where k_i denotes the position index of π in the sequence. We first construct a relative distance matrix D, whose entries are defined as

$$D_{i,j} = |k_i - k_j|$$

This matrix characterizes the absolute positional distances between different occurrences of π along the sequence. Next, a nested sequence of undirected graphs is constructed. The vertex set is given by $\{\pi_{k_1}, \pi_{k_2}, \ldots, \pi_{k_n}\}$. Given a distance filtration threshold d, an undirected edge is added between vertices π_{k_i} and π_{k_j} if

$$|k_i - k_j| \leq d.$$

The weight of the edge is set to $|k_i - k_j|$, yielding a weighted undirected graph denoted by $G(d)$. As the filtration value d increases, a nested sequence of undirected graphs is obtained:

$$G(d_0) \subset G(d_1) \subset G(d_2) \subset \cdots \subset G(d_N).$$

Each graph $G(d_i)$ is then treated as a directed graph by assigning bidirectional arrows to each undirected edge. Persistent homology is computed over the resulting nested sequence of directed graphs. The extracted barcode information, including statistical features of the 0-dimensional, 1-dimensional, and 2-dimensional barcodes, is used as the topological representation of π in the sequence.

This approach not only characterizes the positional distribution of π along the protein sequence, but also captures adjacency patterns and topological structures formed by π at different locations, providing a novel topological perspective for protein sequence analysis.

Feature Extraction Implementation

We construct persistent GLMY homology features (GLMY features) from protein sequences by considering both individual residues (1-mer) and residue pairs (2-mer) to capture topological information. Specifically, we compute GLMY features for all 1-mer and the 20 most frequent 2-mer across the dataset. For each residue π, a nested undirected graph is constructed, and persistent GLMY homology is computed for the three homology dimensions H_0, H_1, H_2 (or 2-dimensional cycles). From the corresponding barcodes, six statistical features are extracted for each homology dimension to form the model input.

For computational efficiency, although there are theoretically $20 \times 20 = 400$ possible 2-mer combinations, we only consider the 20 most frequent 2-mer, as including all combinations would significantly increase computational cost and produce many sparse features, which could hinder model training.

The maximum filtration distance $d_{\max}$ is determined by first estimating the average distances between identical residues using kernel density estimation, followed by a grid search to optimize $d_{\max}$. During graph construction, an edge between two identical k-mers is only added if $d(i,j) \leq d_{\max}$, where $d(i,j)$ denotes the sequence distance between the two mers.

The six statistics extracted from the barcode information are defined as follows. Let ℓ_i denote the lifetime of the i-th bar, N be the total number of bars, and $d_{\max}$ the maximum filtration distance:

1. **Count of bars:** N
2. **Maximum lifetime:** $\max_i \ell_i$
3. **Mean lifetime:** $\frac{1}{N}\sum_{i=1}^{N} \ell_i$
4. **Total lifetime:** $\sum_{i=1}^{N} \ell_i$
5. **Long-lived bars:** $\#\left\{ \ell_i \mid \ell_i > \frac{d_{\max}}{2} \right\}$
6. **Minimum lifetime:** $\min_i \ell_i$

Thus, each 1-mer or 2-mer is represented by an 18-dimensional vector (3 homology dimensions $\times 6$ statistics). At the level of the full protein sequence, the total persistent homology feature dimension is $(20 \text{ 1-mers} + 20 \text{ 2-mers}) \times 18 = 720$.

Finally, we systematically evaluated different strategies for selecting GLMY homology features and used the Macro AUC-PR as the performance metric, as described in Section B.5 of the Methods.

A.1.2 Selection of 1-mer and 2-mer Features

First, we trained the model using only 1-mer features, i.e., extracting topological information solely from individual residues. Subsequently, we incorporated the 20 most frequent 2-mer features observed across all protein sequences. The selected 2-mers are:

AA, AG, AL, AV, EA, EL, GL, GS, HH, LA, LE, LG, LK, LL, LS, LV, SG, SL, VA, VL.

The experimental results are shown in Figure 2**a** in the main text. Each column corresponds to a specific value of the maximum filtration distance parameter, and each row represents a different feature extraction strategy. Overall, across different maximum filtration distance settings, training with only 1-mer features achieved superior predictive performance in the majority of cases (approximately 75%).

These results indicate that 1-mer features possess strong expressive power in capturing the global topological structure and semantic information of protein sequences. The relative positional distribution of individual residues and their global traversal patterns are sufficient to largely represent the overall structural features of the sequence. Consequently, relying solely on 1-mer features yields a stable and discriminative representation.

In comparison, incorporating 2-mer features still outperforms the baseline model without GLMY features, suggesting that the topological information of residue pairs can partially complement local structural features of sequences. However, due to the high sparsity of 2-mer features in real sequences, many feature values are zero, which limits the model's ability to effectively learn their topological patterns. As a result, in most parameter settings, the model with 2-mer features does not surpass the performance obtained using only 1-mer features.

A.1.3 Exploration of Dimensionality for 2-mer Features

During the extraction of persistent GLMY homology features, the Betti numbers of the 2-dimensional homology are mostly zero under the given distance constraints. Therefore, using them directly as features is not ideal. Instead, we chose to use the dimension of the 2-dimensional cycle group as the feature.

In Figure 2**a** in the main text, "1mer+2mer_Z2" refers to using all 1-mer and the 20 most frequent 2-mer features, calculating the 0-dimensional and 1-dimensional persistent GLMY homology as well as the 2-dimensional cycle information, extracting statistical features from the barcodes, and using these as input features to train the model. "1mer+2mer_H2" refers to calculating the 0-dimensional, 1-dimensional, and 2-dimensional persistent GLMY homology of the same 1-mer and 2-mer features, and using the corresponding barcode statistics as input for model training.

The model testing results show that in most cases (62.5%), the performance with "1mer+2mer_H2" is worse than that with "1mer+2mer_Z2". This is primarily because, within the range of the maximum filtration value, the Betti numbers for 2-dimensional paths in the undirected graphs are mostly zero.

Based on the comparative analysis of these experiments, we ultimately selected using only all 1-mer features for constructing persistent GLMY homology sequence topological features. This feature configuration demonstrates more stable and generally superior predictive performance across different filtration parameter settings, while effectively capturing the global topological and semantic information of protein sequences and avoiding feature sparsity and excessive computational complexity.

A.1.4 Selection of Maximum Filtration Distance Parameter

During the construction of persistent GLMY features, the choice of the maximum filtration distance parameter has a critical impact on the extracted topological features. When the maximum filtration distance is too large, as the filtration distance d increases, the resulting graph gradually approaches a complete graph. In this case, the topological features degenerate: the 0-dimensional homology class has only one connected component, and the 1-dimensional and 2-dimensional homology classes vanish, i.e., $\beta_0 = 1, \quad \beta_1 = 0, \quad \beta_2 = 0$. Although this process captures the full evolution of residue (or residue-pair) topology with increasing distance, it significantly increases computational complexity, and the overly trivial topological representation is not conducive to effective model learning and prediction. Conversely, if the maximum filtration distance is too small, edges between different *mer*s are difficult to form, and the 1-dimensional and 2-dimensional homology classes rarely change, failing to capture the topological relationships among *mer*s in the sequence.

To address this, we first constructed a statistic that reflects the "average residue distance" in the sequence and used it as a reference for selecting the maximum filtration distance. Specifically, for each protein sequence, for each residue type, we calculated the distances between each residue and its nearest neighboring occurrence, then took the average, yielding 20 average distance values per sequence corresponding to the 20 residue types. These averages were then aggregated across all protein sequences, and a kernel density estimation (KDE) was performed to model the probability density distribution. The peak of this distribution, representing the main concentration of the data, was found to be approximately 11.8859, as shown in Figure 2**b** in the main text.

Based on this statistic, we conducted a finer grid search over maximum filtration distances ranging from 3 to 18 to systematically evaluate the impact of different parameter settings on model performance. The experiments covered multiple feature configurations, including: using only 1-mer features; incorporating 2-mer features in addition to 1-mer and considering 0 to 1-dimensional as well as 2-dimensional cycle group; and various combinations of 0 to 2-dimensional homology for 2-mer features. The corresponding model performance results are summarized in Figure 2**a** in the main text. Experimental results indicate that

a maximum filtration distance of 8 yields the best predictive performance. Accordingly, we selected this configuration as the final parameter for extracting persistent GLMY homology features.

These results demonstrate that the choice of maximum filtration distance has a significant influence on model performance. Setting the maximum filtration distance to 8 achieves optimal performance, indicating that local interactions among residues within approximately 8 positions are most relevant for functional prediction. Distances that are too large (> 12) lead to oversimplified topological features, while distances that are too small (< 5) fail to capture sufficient sequence topological information.

A.2 GLMY homology

GLMY homology (also known as path homology) was proposed by Professor Shing-Tung Yau and his collaborators in 2012[1]. Its core innovation is to construct path complexes directly from directed graph structures, forming chain complexes through ∂-invariant paths, and subsequently defining homology groups. This allows algebraic topology tools to be naturally applied to directed networks while preserving intrinsic directional information. Moreover, since undirected networks can be considered a special case of directed networks (e.g., by replacing each undirected edge with a pair of bidirectional edges), GLMY homology can also be used to extract structural and geometric features from such data. But in undirected graphs, the presence of numerous symmetric edges often causes standard GLMY homology to collapse cycles formed by symmetric edge pairs. To address this issue, we employ primitive GLMY homology, which is better suited for GLMY-homological analysis of undirected graphs. This framework preserves cycle information that would otherwise be eliminated by symmetry, following the approach described in [2]. Below we provide the formal definition of this GLMY homology.

Let $G = (V, E)$ be a directed graph. An elementary **allowed** n**-path** is a vertex sequence $i_0 i_1 ... i_n$ such that for each consecutive pair of vertices i_k and i_{k+1}, there exists a directed edge $i_k \to i_{k+1}$ in G. Let $\mathbb{Z}$ be the coefficient ring. The free Abelian group generated by all elementary allowed n-paths in G is denoted as $\mathcal{A}_n(G)$, i.e.,

$$\mathcal{A}_n(G) = \langle e_{i_0 i_1 ... i_n} \mid i_0 i_1 ... i_n \text{ is an allowed path} \rangle.$$

An elementary allowed path $i_0 i_1 ... i_n$ in $\mathcal{A}_n(G)$ is denoted by $e_{i_0 i_1 ... i_n}$. Elementary allowed paths of length n form the generators of $\mathcal{A}_n(G)$.

For each elementary allowed path $e_{i_0 i_1 ... i_n}$ in $\mathcal{A}_n(G)$, define the boundary operator:

$$\partial_n e_{i_0 ... i_n} = \sum_{q=0}^{n} (-1)^q e_{i_0 ... \widehat{i_q} ... i_n},$$

and define $\partial_0(e_{i_0}) = 0$ when $n = 0$. The operator ∂_n is called the n-dimensional boundary operator.

In general, applying the boundary operator to an elementary allowed path of length n does not necessarily yield an allowed path. Therefore, we introduce the subgroup of $\mathcal{A}_n(G)$ consisting of ∂-invariant paths.

Define

$$\Omega_n(G) = \{\nu \in \mathcal{A}_n(G) \mid \partial_n \nu \in \mathcal{A}_{n-1}(G)\},$$

called the ∂-invariant path space.

For $\Omega_n(G)$, we have $\partial_n \Omega_n(G) \subseteq \Omega_{n-1}(G)$ and $\partial_{n-1} \circ \partial_n = 0$. This forms a chain complex:

$$0 \xleftarrow{\partial_0} \Omega_0(G) \xleftarrow{\partial_1} \Omega_1(G) \xleftarrow{\partial_2} \Omega_2(G) \xleftarrow{\partial} \cdots .\Omega_{n-1}(G) \xleftarrow{\partial_n} \Omega_n(G) \xleftarrow{\partial} \cdots$$

The n-th GLMY homology group of a directed graph G is defined as

$$H_n(G) = \ker(\partial_n : \Omega_n \to \Omega_{n-1}) / \operatorname{im}(\partial_{n+1} : \Omega_{n+1} \to \Omega_n).$$

The dimension of the homology group $H_n(G)$ is denoted by β_n, called the n-th Betti number of G.

GLMY homology of a directed graph can detect structural information within the graph:

- β_0: the number of connected components in the directed graph;
- β_1: the number of linearly independent 1-dimensional cycles formed by 1-paths that cannot be contracted or decomposed into smaller directed paths;
- β_n: the number of linearly independent n-dimensional cycles that cannot be expressed as boundaries of $(n+1)$-paths, i.e., the number of n-dimensional cavities generated by n-paths in G.

For directed graphs with relatively simple structure, higher-dimensional homology groups often vanish, while the corresponding cycle groups $\ker(\partial_n)$ may remain nontrivial. In some cases, we may use the dimension c_n of the cycle group Z_n in place of the Betti number β_n to characterize structural features of graphs. In particular, c_2 represents the dimension of the space of two-dimensional cycles, i.e., the number of chains generated by 2-paths in the directed graph whose boundaries vanish under the boundary operator.

A.2.1 Persistent Homology and Barcodes

Persistent homology considers the evolution of homology classes or Betti numbers along a sequence of nested directed graphs obtained according to a certain filtration.

Given a sequence of nested directed graphs

$$G_0 \subset G_1 \subset G_2 \subset \cdots \subset G_N = G,$$

for each $n \geq 0$, we obtain a sequence of GLMY homology groups

$$H_n(G_0) \longrightarrow H_n(G_1) \longrightarrow H_n(G_2) \longrightarrow \cdots \longrightarrow H_n(G_N).$$

The homomorphisms between consecutive homology groups are induced by the inclusion maps. If a homology class is born at time p and dies at time q (due to the addition of higher-dimensional paths whose boundary exactly forms a homology class, causing the disappearance of an existing class), we call p and q the birth and death times of this homology class. Plotting a horizontal line from p to q on the coordinate plane gives an n-dimensional barcode, and $q-p$ is called the persistence of the homology class. Naturally, a homology class may never die after its creation; in this case, the barcode is a ray (p, ∞) with infinite persistence. Plotting all n-dimensional barcodes on the same plane yields the persistent barcode for the sequence of nested directed graphs. From the persistent barcode, one can observe how the structure of the directed graph changes as the filtration scale varies.

For each residue or residue pair in a protein sequence, denoted by the letter π, we extract the positions of π in the sequence and label them as

$$\pi_{k_1}, \pi_{k_2}, \cdots, \pi_{k_n},$$

constructing a pairwise distance matrix D where

$$D_{i,j} = |k_i - k_j|.$$

Using $\pi_{k_1}, \pi_{k_2}, \cdots, \pi_{k_n}$ as the vertex set, we add an undirected edge between π_{k_i} and π_{k_j} whenever their distance is less than or equal to a given distance filtration value d, and assign the edge a weight of $|k_i - k_j|$. This yields the weighted undirected graph $\mathbf{G}(d)$ at filtration d. As d increases, we obtain a nested sequence of undirected graphs:

$$\mathbf{G}(d_0) \subset \mathbf{G}(d_1) \subset \mathbf{G}(d_2) \subset \cdots \subset \mathbf{G}(d_N).$$

Treating each $\mathbf{G}(d_i)$ as a directed graph, we compute the persistent GLMY homology of the nested sequence of directed graphs. The extracted barcode information (e.g., 0-dimensional, 1-dimensional, and 2-dimensional barcodes: maximum lifetime, mean lifetime, total lifetime, number of long-lived bars, minimum lifetime) is used as features for π in the sequence. These features not only capture the positional information of π within the sequence but also reflect the adjacency patterns of π at different positions.

In the dashed box of Figure 1**A** in the main text, as an example, we construct a nested sequence of persistent undirected graphs for the letter P in a segment of a protein sequence, and present its 0-2-dimensional persistent barcodes.

A.2.2 Feature Extraction

In GLMYsymm, we extract 1-mer GLMY homology features from protein sequences. The maximum filtration distance is set to $d_{\max} = 8$, and we compute the following:

- 0-dimensional and 1-dimensional persistent GLMY homology group;

- 2-dimensional persistent cycle group.

For each dimension, we compute the following barcode statistics: count, max_life, mean_life, total_life, long_count ($> d_{\max}/2$), min_life. Since there are 6 statistics per homology dimension and 3 dimensions (0D, 1D, 2D), this results in $3 \times 6 = 18$ features per 1-mer. With 20 amino acid types in a protein sequence, the total feature vector dimension for each sequence is $18 \times 20 = 360$.

A.3 Differential Scoring

We performed a systematic statistical analysis of 1-mer persistent GLMY features to investigate whether there exist stable and interpretable 1-mer topological differences among different protein symmetry classes.

First, protein sequences were categorized according to their structural symmetry. For each sequence, under the 1-mer setting, we constructed a nested sequence of undirected graphs based on the relative positional distances between residues, with the maximum filtration distance set to 8, and computed the persistent GLMY homology. For each symmetry class, we aggregated the 1-dimensional persistent diagrams (β_1) of all sequences in that class, which record the birth and death times of 1-dimensional homology classes induced by each 1-mer in the sequence.

To characterize the topological difference of a given 1-mer within a specific symmetry class relative to other classes, we introduce a scoring function based on persistent diagrams.

For a fixed symmetry class c and a 1-mer (letter) π, we aggregate all 1-dimensional persistent diagrams of π across sequences in this class, denoted as

$$PD_{c,\pi}^{(\beta_1)} = \{(b_1, d_1), (b_2, d_2), \ldots, (b_N, d_N)\},$$

where $b_i \in \{0, 1, \ldots, 8\}$ indicates the birth time and $d_i \in \{1, \ldots, 9\}$ indicates the death time of the homology class. Note that, if a homology class does not disappear within the maximum filtration distance 8, its death time is set as $d_i = 9$.

Based on this, we define a discrete birth-scale axis $b = 0, \ldots, 8$, and define the 1-dimensional homology generation distribution vector of letter π in symmetry class c as

$$\mathbf{v}_{c,\pi}^{(\beta_1)} = \big(n_{c,\pi}(b = 1), n_{c,\pi}(b = 2), \ldots, n_{c,\pi}(b = 8)\big),$$

where

$$n_{c,\pi}(b = k) = \#\big\{(b_i, d_i) \in PD_{c,\pi}^{(\beta_1)} : b_i = k\big\}.$$

This vector characterizes the distribution of 1-dimensional homology classes induced by π at different scales within the maximum filtration distance in symmetry class c.

Let $\mathcal{C}$ denote the set of all symmetry classes. For a fixed π, we define its mean generation distribution vector across all non-c classes as

$$\bar{\mathbf{v}}^{(\beta_1)}_{others,\pi} = \frac{1}{|\mathcal{C}|-1} \sum_{c' \in \mathcal{C}\setminus\{c\}} \mathbf{v}^{(\beta_1)}_{c',\pi}.$$

In this work, we consider 15 symmetry classes, so $|\mathcal{C}|-1 = 14$. This mean vector can be interpreted as the "typical background" of the 1-dimensional homology distribution of letter π across non-c symmetry classes.

Based on the above definitions, we introduce the following differential scoring function:

$$\text{score}(c,\pi) = \left\| \mathbf{v}^{(\beta_1)}_{c,\pi} - \bar{\mathbf{v}}^{(\beta_1)}_{others,\pi} \right\|,$$

where $\|\cdot\|$ denotes the Euclidean norm. This score measures the deviation of the 1-dimensional homology distribution induced by letter π in symmetry class c relative to the average distribution in other classes. A larger score(c,π) indicates that the 1-mer topology corresponding to this letter is more specific to the symmetry class c.

A.4 ESM2 Features

The protein language model ESM2 is currently a state-of-the-art pre-trained model for extracting protein sequence representations, capable of learning structural, functional, and evolutionary information directly from amino acid sequences. The core of the ESM2 approach is based on the Masked Language Modeling (MLM) task, which is a self-supervised learning objective built upon the Transformer architecture. Importantly, ESM2 does not require multiple sequence alignments (MSA) to capture evolutionary conservation information during training. At present, many studies [3–5] leverage ESM2 embeddings for downstream tasks.

In this study, we used the `esm2_t33_650M_UR50D` model [6] to generate embeddings for each protein sequence. For a sequence of length L, the initial embedding is a matrix of dimension $L \times 1280$, representing per-residue features. We then performed average pooling along the residue dimension to obtain a fixed-length vector of dimension 1×1280 for each sequence. Finally, this vector is passed through a multi-layer perceptron (MLP) to reduce its dimension to 1×256, which is subsequently concatenated with the GLMY homology features.

A.5 Macro AUC-PR

The Area Under the Precision-Recall Curve (Macro AUC-PR) is an important evaluation metric for multi-class tasks. It is computed by converting the multi-class problem into multiple binary classification problems. Specifically, for each class t, we treat it as the positive class and all other classes as negative, and then calculate the Precision and Recall values for that class:

$$\text{Precision}_t = \frac{TP_t}{TP_t + FP_t} \tag{1}$$

$$\text{Recall}_t = \frac{TP_t}{TP_t + FN_t} \tag{2}$$

where TP_t, FP_t, and FN_t denote true positives, false positives, and false negatives for class t, respectively. The Area Under the Precision-Recall Curve (AUC-PR) for class t can be approximated using discrete points along the curve as:

$$\text{AUC-PR}_t \approx \sum_{i=1}^{N-1} (\text{Recall}_{t,i+1} - \text{Recall}_{t,i}) \cdot \text{Precision}_{t,i+1} \tag{3}$$

where N is the number of threshold points and $(\text{Recall}_{t,i}, \text{Precision}_{t,i})$ are the Precision-Recall values for class t at the i-th threshold. Finally, the Macro AUC-PR is obtained by averaging over all C classes:

$$\text{Macro AUC-PR} = \frac{1}{C} \sum_{t=1}^{C} \text{AUC-PR}_t \tag{4}$$

Since the dataset is highly imbalanced across different classes, Macro AUC-PR is chosen to evaluate the model's predictive capability. In cases where the model is biased towards predicting majority classes, overall accuracy might appear high, but the performance on minority classes can be poor. Macro AUC-PR treats all classes equally, taking both precision and recall into account, making it more suitable than single accuracy for imbalanced data.

Moreover, unlike Micro AUC-PR, Macro AUC-PR is not dominated by the majority classes. Therefore, it provides a fair and robust metric to evaluate and compare model performance across all classes.

A.6 Loss Function

The GLMYsymm model uses the Focal Loss (FL) as its loss function, which is computed as follows. For class t, its Effective Number weight w_t is defined as:

$$w_t = \frac{1 - \beta^{n_t}}{1 - \beta}$$

where n_t denotes the number of samples in class t, and $\beta \in (0, 1)$ is a hyperparameter controlling the degree of weight disparity. A larger β results in more pronounced weight differences, giving greater attention to minority classes. The weights are then normalized:

$$\alpha_t = \frac{w_t}{\sum_i w_i}$$

The Focal Loss is expressed as:

$$FL(p_t) = -\alpha_t \, (1 - p_t)^\gamma \, \log(p_t)$$

where p_t is the predicted probability for the true class, α_t is the normalized class weight based on the effective number of samples for class t, and γ is the focusing parameter. In this study, we set $\gamma = 3$ to emphasize hard-to-classify samples.

In the extremely imbalanced scenarios considered in this work, Focal Loss demonstrates strong adaptability and effectively enhances the model's discriminative ability on minority classes.

Appendix B Performance Analysis of the Model

We analyzed the predictive performance of GLMYsymm on the model's test set. The results show substantial differences in performance across different symmetry orders. Overall, low-order symmetry types (C1–C5 and D2–D3) achieve better predictive performance than high-order symmetry types. Specifically, the average Macro AUC-PR for low-order symmetric proteins reaches 0.631, whereas the average Macro AUC-PR for high-order symmetric proteins is 0.557. These results indicate that GLMYsymm demonstrates more stable predictive capability in identifying low-order symmetric structures, while there remains room for improvement in predicting high-order symmetric structures.

In fact, within existing protein databases, low-order symmetric proteins are far more abundant than high-order symmetric proteins, and their structural types are also more diverse. A larger number of data samples provides the model with more learnable structural patterns, thereby enhancing its predictive performance for the corresponding categories. In addition, high-order symmetric structures often exhibit more complex spatial organizations and stricter geometric constraints, which further increases the difficulty of recognition. Therefore, differences in data scale and structural complexity may be important factors contributing to the imbalance in predictive performance across different symmetry orders.

Appendix C Ablation Study of the Training-Set Oversampling Strategy

To evaluate the impact of the training-set oversampling strategy on class-imbalanced classification, we conducted a controlled experiment without oversampling while keeping the data split, model architecture, and hyperparameter settings identical. The validation and test sets retained their original class distributions.

The overall and per-class performance comparisons are summarized in Table S1, while the corresponding normalized confusion matrices are shown in Figure S1. As shown in Table S1, oversampling substantially improved the Macro Average Precision, Recall, and F1-score. Consistent with the results reported in the main text, the oversampled model also achieved considerably higher Balanced Accuracy, Macro-F1, and Macro AUC-PR values than the model trained without oversampling. Although the non-oversampled model obtained a slightly

higher overall Accuracy, the class-wise metrics indicate that it was strongly biased toward majority symmetry categories.

The per-class results in Table S1 show that the rarest symmetry categories in the test set (C10–C17, C7–C9, D5, D6–D8, H and T) were almost completely unrecognized without oversampling, with Precision, Recall, and F1-score values close to zero. After oversampling, all of these categories achieved non-zero Precision, Recall, and F1-score values. In particular, C10–C17, C7–C9, D5, and T exhibited substantial improvements across all three metrics, indicating that the model became capable of learning discriminative features for these underrepresented symmetry categories. Although the improvement for D6–D8 remained relatively limited due to its extremely small sample size, the model nevertheless acquired a measurable predictive capability for this category. In addition, several medium-sized minority categories, such as C4, C5, D3, and D4, also benefited from oversampling.

The confusion matrices in Figure S1 provide further evidence of this effect. The left panel (without oversampling) shows a pronounced majority-class bias, where many minority-class samples are incorrectly assigned to dominant categories such as C1 and C2. In contrast, the right panel (with oversampling) exhibits a considerably more balanced prediction distribution and stronger diagonal elements for several minority and high-order symmetry categories, indicating improved recognition capability.

Overall, the results presented in Table S1 and Figure S1 demonstrate that training-set oversampling effectively mitigates the bias caused by class imbalance. At the cost of only a minor reduction in overall Accuracy, it substantially improves minority-category recognition and class-balanced performance. The analysis further reveals that low-order and well-represented symmetry categories can be predicted relatively reliably, whereas several rare and high-order symmetry categories remain challenging due to limited training samples. Therefore, the oversampling strategy was retained in the final GLMYsymm model.

Appendix D Model Generalization

To validate the generalization capability of GLMYsymm, we conducted predictions on several proteins of different types that were not included in the dataset and obtained favorable results. The following presents representative examples.

Homologous Soluble Protein

The Crystal structure of an extracellular domain fragment of human BAFF is a homologous soluble protein (PDB ID: 1KD7). This protein was not included in the dataset used to train the model. The prediction result of GLMYsymm is consistent with the experimentally determined symmetry type recorded in the database, both indicating C3 symmetry. The structural visualization is shown in Figure 5**a** in the main text.

Heterologous Insoluble Protein

The Crystal structure of BamB and BamA P3–5 complex from E. coli is a heterologous insoluble protein (PDB ID: 4XGA). As a heterologous complex, it

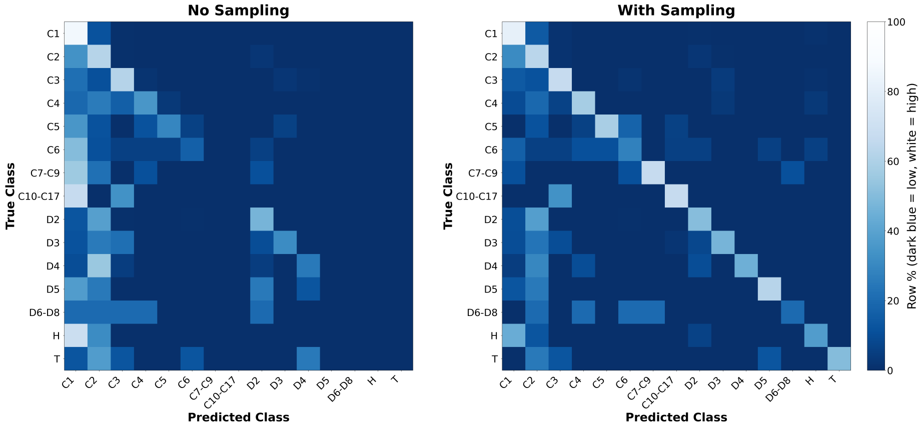

Figure S1: Normalized confusion matrices of GLMYsymm on the test set without oversampling (left) and with oversampling (right). Each row is normalized such that the entries represent the proportion of samples from a given true class that are predicted as different classes. Compared with the model trained without oversampling, the oversampled model exhibits noticeably stronger diagonal elements for several minority symmetry classes, including C10–C17, C7–C9, D5, D6–D8, H, and T, indicating improved recognition capability, while the classification performance on majority classes remains largely unchanged.

Table S1: Comparison of per-class Precision, Recall, and F1-score with and without training-set oversampling. Positive Δ values indicate performance improvements introduced by oversampling.

Symmetry Class	Test Samples	Precision			Recall			F1-score		
		No Sampling	Sampling	Δ	No Sampling	Sampling	Δ	No Sampling	Sampling	Δ
C1	2171	0.8034	0.8210	+0.0176	0.8696	0.8158	-0.0538	0.8352	0.8184	-0.0168
C2	1067	0.6351	0.6125	-0.0227	0.6232	0.6354	+0.0122	0.6291	0.6237	-0.0054
C3	124	0.6364	0.6014	-0.0349	0.6210	0.6694	+0.0484	0.6286	0.6336	+0.0050
C4	31	0.5500	0.6207	+0.0707	0.3548	0.5806	+0.2258	0.4314	0.6000	+0.1686
C5	17	0.5556	0.5556	0.0000	0.2941	0.5882	+0.2941	0.3846	0.5714	+0.1868
C6	18	0.4286	0.2273	-0.2013	0.1667	0.2778	+0.1111	0.2400	0.2500	+0.0100
C7–C9	9	0.0000	0.5000	+0.5000	0.0000	0.6667	+0.6667	0.0000	0.5714	+0.5714
C10–C17	3	0.0000	0.2857	+0.2857	0.0000	0.6667	+0.6667	0.0000	0.4000	+0.4000
D2	164	0.6311	0.6434	+0.0123	0.4695	0.5061	+0.0366	0.5385	0.5666	+0.0281
D3	51	0.6400	0.6316	-0.0084	0.3137	0.4706	+0.1569	0.4211	0.5393	+0.1182
D4	20	0.5556	0.9000	+0.3444	0.2500	0.4500	+0.2000	0.3448	0.6000	+0.2552
D5	8	0.0000	0.5556	+0.5556	0.0000	0.6250	+0.6250	0.0000	0.5882	+0.5882
D6–D8	5	0.0000	0.2500	+0.2500	0.0000	0.2000	+0.2000	0.0000	0.2222	+0.2222
H	16	0.0000	0.2143	+0.2143	0.0000	0.3750	+0.3750	0.0000	0.2727	+0.2727
T	8	0.0000	1.0000	+1.0000	0.0000	0.5000	+0.5000	0.0000	0.6667	+0.6667
Macro Avg	–	0.3624	0.5613	+0.1989	0.2640	0.5350	+0.2710	0.2970	0.5280	+0.2310

Precision, Recall, and F1-score are reported for both the no-sampling and oversampling settings. Δ represents the performance change introduced by oversampling and is calculated as Sampling − No Sampling. Test Samples indicates the number of test samples belonging to each symmetry category.

was not considered during data selection. Since GLMYsymm is designed to predict symmetry types based on single-chain sequence information, the input consists of individual sequences. To evaluate the model's generalization ability, we treated protein 4XGA (Global Stoichiometry: A1B1) as two separate sequences for prediction. The predicted symmetry type for both chains was C1, indicating no symmetry-related assembly and a monomeric state in the structure. This result is consistent with the actual structure, demonstrating that GLMYsymm can not only predict symmetry types but also assist in stoichiometry-related analysis. The structure is shown in Figure 5**b** in the main text.

Multi-Label Protein

In addition, we evaluated GLMYsymm on proteins capable of forming multiple symmetry types. For example, the Structure of CYP119 with T213A and C317H mutations (PDB ID: 5BV5) can naturally adopt C1, C2, and D2 symmetry states. Among the predicted results, the top three most probable symmetry types were C1, C2, and D2, which are consistent with the experimentally observed possibilities. This indicates that the multiple predicted symmetry types provided by GLMYsymm can all represent plausible structural states that the protein may adopt. The structure is shown in Figure 5**c** in the main text.

In summary, the proposed GLMYsymm model not only performs well on the test dataset but also demonstrates strong performance on independent data outside the dataset. It shows accurate predictive capability across different protein types, including soluble and insoluble proteins, heterologous complexes, and proteins capable of forming multiple symmetry types, thereby indicating that GLMYsymm possesses strong generalization ability.

Appendix E Training Configuration and Experimental Environment

To ensure reproducibility, the detailed training configuration and experimental environment are summarized as follows. Model training employed an early stopping strategy based on validation Macro AUC-PR, with a patience of 30 epochs and a maximum training budget of 200 epochs; the top three checkpoints with the best validation performance were retained for model selection. The ESM2 sequence representations and path homology topological features were extracted offline prior to training and remained fixed throughout optimization. All parameters in the downstream QCformer architecture, including the feature fusion MLP module (approximately 1.53 million trainable parameters), were optimized without applying any layer-freezing strategy.

Optimization was performed using the AdamW optimizer with an initial learning rate of 2×10^{-4} and a weight decay coefficient of 2×10^{-4}. A `CosineAnnealingLR` scheduler was adopted to gradually decrease the learning rate, with $T_{\max} = 200$ and a minimum learning rate of $\eta_{\min} = 2 \times 10^{-7}$.

All experiments were conducted on a single NVIDIA GH200 accelerator equipped with an ARM Neoverse-V2 72-core CPU and 573 GB system memory.

The software environment was based on `PyTorch 2.12.0` with `CUDA 13.0` support. The complete configuration file is provided in `config/protein_classifier_config.yaml`, and the corresponding training implementation is available in `train_protein_improved.py` within the open-source repository.

Statistical Analysis of Data Distributions

Table S2: This table summarizes all symmetry types and their corresponding sample sizes used in GLMYsymm, including the total number of samples for each symmetry type, the number of training samples before sampling, the number of training samples after sampling, as well as the numbers of validation and test samples.

Type \ Count	Total Samples	Training Set	Post-sampling Count	Validation Set	Test Set
C1	21716	17372	10423	2173	2173
C2	10677	8541	8541	1067	1069
C3	1244	995	4975	124	125
C4	313	250	5000	31	32
C5	170	136	2720	17	17
C6	187	149	2980	18	20
C7-C9	97	77	1540	9	11
C10-C17	73	55	1100	8	10
D2	1642	1313	6565	164	165
D3	512	409	8180	51	52
D4	202	161	3220	20	21
D5	81	64	1280	8	9
D6-D8	74	58	1160	6	10
H	169	135	2700	16	16
T	86	68	1360	8	10

Table S3: Label merging strategy for rare symmetry classes and their sample distributions before merging.

Merged category	Original classes (sample counts)	Total samples
C7–C9	C7 (53), C8 (30),<br>C9 (14)	97
D6–D8	D6 (43), D7 (19),<br>D8 (12)	74
C10–C17	C10 (11), C11 (14), C12 (27), C13 (5),<br>C14 (6), C15 (7), C16 (2), C17 (1)	73

Note: To alleviate severe class imbalance caused by the extremely limited number of samples in several high-order symmetry classes, adjacent symmetry orders were merged into interval-based categories. Specifically, C7–C9, D6–D8, and C10–C17 were merged into three categories. Numbers in parentheses indicate the sample counts of the original classes before merging, while the last column reports the total number of samples in the merged category.

Table S4: Different residues and their score values across all symmetry categories. Each row in the table corresponds to a distinct symmetry class, and each column corresponds to a different residue type. A larger score indicates a greater degree of topological dissimilarity of that residue within the given symmetry class compared to its topological structures in other symmetry classes. Because the full table is too large, it is divided into subtables.

class	A	C	D	E	F	G
C1	0.0243725535694096	0.07636390336240112	0.012538406386221716	0.011105683620018396	0.019160168464156316	0.01913594103441422
C10-C17	0.047714256640637095	0.19013877797671816	0.04571089700464352	0.048769967122593	0.11424428346954546	0.03413292017910934
C2	0.00810589947845819	0.03058946940952808	0.016048178531192583	0.012379373773630622	0.015608120349752945	0.016710240165479173
C3	0.015250385169263485	0.07502944122041136	0.022585069074975723	0.017312076525931783	0.02569017361975159	0.0226998665705106
C4	0.035390429715300466	0.11789545361419888	0.023446795680043764	0.030897320883504366	0.0588373261973732	0.043654383740480356
C5	0.04077273652991752	0.11875752956124254	0.05245798014739037	0.050310946128934546	0.06553695886813282	0.03445612055100888
C6	0.02949723235605147	0.15826323212262838	0.04469146556970698	0.030129633195364918	0.03769991247774072	0.03753388539347493
C7-C9	0.03784274795754262	0.2745038313661548	0.05217322716357158	0.03227695852504492	0.09928546500037609	0.061837897802654085
D2	0.020786720244319032	0.0797450014873652	0.02134803219673385	0.02805317644982305	0.03972917416834629	0.02383374077545275
D3	0.020642849920682597	0.07158155661117853	0.023003095135862905	0.026657596433632583	0.03503591426123092	0.027244962240711566
D4	0.06422033563134548	0.14860144545711287	0.06172952534094924	0.04884606852471051	0.06429975404429451	0.06240191245662077
D5	0.05679774597585934	0.18331830791836623	0.0665073803915212	0.0600376368513088	0.08218032023183253	0.05234003889650833
D6-D8	0.07414242367273866	0.1536313835284895	0.07219062784903683	0.04081252441056058	0.07680946386818126	0.04868886203555152
H	0.03585130573004501	0.43643794349877507	0.04682276769838636	0.038049168167077776	0.08337507187779769	0.04717474579144501
T	0.04048103738963272	0.14325700144991552	0.0658889530294897	0.06207717999240485	0.09224026186372775	0.04881496840621923

class	H	I	K	L	M	N
C1	0.031246358721686483	0.02097570407921319	0.018363976770708515	0.010454321973712025	0.022677594888145546	0.021336262904715627
C10-C17	0.1852385645466497	0.047621046120467816	0.06434690059057246	0.04038133075586052	0.11290332175344507	0.05521208791954629
C2	0.02699149727349061	0.018384120328878303	0.010542266396272173	0.010481191386065425	0.031004388627678778	0.02395115205660627
C3	0.07234788995284938	0.0314721803090864	0.02998669758684334	0.0260838595385283	0.05713150772963865	0.0391563012418232
C4	0.11443054169865612	0.031989666069201006	0.04837073301217043	0.05560323860852256	0.0718094584693434	0.043510890500013055
C5	0.08054475126140871	0.049307589491802535	0.04199573450224553	0.04982679690877138	0.08341959309246282	0.03929712846786499
C6	0.10485609671437142	0.033567248858043075	0.038353188672525226	0.023615004816663787	0.10173083943857485	0.036058326783033175
C7-C9	0.14883411964684742	0.05691178696631334	0.04145029414239693	0.0393172821420366	0.14861879540599052	0.07762417377056222
D2	0.0390019313431714	0.029651021557206617	0.031585012381713976	0.029733114939249433	0.04553121694332631	0.03787542972161932
D3	0.04797247260513795	0.02831757384219889	0.026283647788324874	0.02892057079412385	0.05406852160378423	0.03298522393088172
D4	0.08271249967703716	0.05109048606789949	0.05906461830949585	0.051566698940943075	0.08284557792307777	0.0748683623371542
D5	0.11855979665311733	0.07080027808827628	0.06918653410902775	0.04486949521416613	0.12682171281744595	0.10866571247698434
D6-D8	0.18403882528730234	0.054296151791275137	0.054957961615736954	0.055087782198176886	0.13088989706336002	0.055277511026367944
H	0.09521158398829606	0.037004371895621266	0.054738717434888645	0.04081500173611381	0.08782857657327657	0.04426997703296047
T	0.14175378135397596	0.06160617052441155	0.04745806036424225	0.04760668371064781	0.13319622669593667	0.0987145230546337

class	P	Q	R	S	T	V
C1	0.02231045886963143	0.0171593043306434	0.013924782473188686	0.026495911576850904	0.012767428432644957	0.020379797568604436
C10-C17	0.05723831064035973	0.06201686555754205	0.06215242485967263	0.046254656749753646	0.05568222508083481	0.03875542393271159
C2	0.025290805786646966	0.019645416697177779	0.014583805959155283	0.0178567402762969	0.02028765310200069	0.014918832645387075
C3	0.03606130983130615	0.0299859302798756	0.02176162315238356	0.024776297608279683	0.024602421968729183	0.02377626050113022
C4	0.038841149828924525	0.04426127356837469	0.04300645926215858	0.03348477149786307	0.028717305767108844	0.027516515402567575
C5	0.056065278756237805	0.07071036582202689	0.05937262318860717	0.039585160681721514	0.055162108969466765	0.043327119827525164
C6	0.05856523352758121	0.04969597542554554	0.04549114205088008	0.04323490449645667	0.05172089278392452	0.022708901789246515
C7-C9	0.09790847121458908	0.06840118860696721	0.04909062926040863	0.05203850390040594	0.07340482863511565	0.045643900670191515
D2	0.04117104799679362	0.03043766229251537	0.022234591100496574	0.03023640431601871	0.0318939976681185	0.025207609040209616
D3	0.04257488573470463	0.03064600923803577	0.026320133930666557	0.030027232272258574	0.04602053260189317	0.024893223930214772
D4	0.08934968555049978	0.0672780482115481	0.03493550057331324	0.05150393809558299	0.0825427424045408	0.047611830218730736
D5	0.08824774475703873	0.06863344906013887	0.05775401082501176	0.05883708129360404	0.04871316774818023	0.0481207730122508 6
D6-D8	0.09108168389876264	0.06782909800393098	0.05968182299006457	0.053332581144816	0.066253963929949 26	0.050594748756970544
H	0.21602422935077253	0.06514925883181817	0.054592573196162814	0.05776765709278816	0.05814765108980114	0.03494373059209679
T	0.07289927140466126	0.05931634896723955	0.09656747691692272	0.06299768936294943	0.06397350479733095	0.06629983501349615

class	W	Y
C1	0.04617173458065779	0.03321404712735813
C10-C17	0.20641299735433033	0.1098596910202324
C2	0.04554509759508737	0.03311676664419363
C3	0.07329797585658404	0.04297104339546969
C4	0.1339105990343291	0.06730337477959503
C5	0.13619415756087785	0.08252669542531553
C6	0.10830050684603641	0.04823261510528884
C7-C9	0.15457271396876604	0.07375599419167378
D2	0.0768589015720006	0.03563529315741223
D3	0.08926866793673179	0.05482000987810964
D4	0.07718284231847075	0.05693540722484735
D5	0.25706345304156564	0.05340023276058678
D6-D8	0.24556129766237603	0.09781309925657324
H	0.2511850723262202	0.08586459496378657
T	0.19985372422848993	0.12563075347921082

References

[1] Alexander Grigor'yan, Yong Lin, Yuri Muranov, and Shing-Tung Yau. Homologies of path complexes and digraphs. arXiv preprint arXiv:1207.2834, 2012.

[2] Jingyan Li, Yuri Muranov, Jie Wu, and Shing-Tung Yau. Primitive path homology. Data Analytics and Topology, 1(1):1–15, 2025.

[3] Lijuan Hou, Hanyan Qin, Xiankun Zhang, and Yiying Zhang. Protein function prediction based on the pretrained language model esm2 and graph convolutional networks. In 2024 IEEE International Symposium on Parallel and Distributed Processing with Applications (ISPA), pages 1776–1781. IEEE, 2024.

[4] Hong-Qi Zhang, Shang-Hua Liu, Jun-Wen Yu, Rui Li, Dong-Xin Ye, Yan-Ting Jin, Cheng-Bing Huang, and Ke-Jun Deng. Drugpred: An ensemble learning model based on esm2 for predicting potential druggable proteins. Future Generation Computer Systems, 170:107801, 2025.

[5] Zeyu Luo, Rui Wang, Yawen Sun, Junhao Liu, Zongqing Chen, and Yu-Juan Zhang. Interpretable feature extraction and dimensionality reduction in esm2 for protein localization prediction. Briefings in Bioinformatics, 25(2):bbad534, 2024.

[6] Zeming Lin, Halil Akin, Roshan Rao, Brian Hie, Zhongkai Zhu, Wenting Lu, Nikita Smetanin, Allan dos Santos Costa, Maryam Fazel-Zarandi, Tom Sercu, Sal Candido, et al. Language models of protein sequences at the scale of evolution enable accurate structure prediction. bioRxiv, 2022.
